# Gunshot Wounds Produced during the Loading of Artillery

**Published:** 1861-08

**Authors:** 


### GUNSHOT WOUNDS PRODUCED DURING THE LOADING OF ARTILLERY.

Dr. Cortese relates five cases of gunshot wounds received by artillerymen while engaged in loading their guns, and gives the following summary of his observation: 1. No other blow of a projectile imparts like the present so great an amount of commotion to the entire limb. The state of muscular contraction prevailing at the time constitutes a kind of solidarity between the hand, forearm, and arm, which is the chief and necessary cause of this commotion. 2. This circumstance complets the surgeon to direct his attention to the entire limb, whatever amount of lession may be manifest in the hand. A neglect of this precept may lead to gangrene gaining possession of a large portion of the limb, or to a generalized suppuration, while a diminished power of reaction in the injured parts may give rise to purulent infection, or render useless recourse to amputation. 3. In all those cases in which the hand is severely torn, its disarticulation, or even the amputation of the forearm, is insufficient to secure recovery. The surgeon's knife penetrates into infiltrated tissues, more or less destroyed in their intimate structure in consequence of the concussion they have been subjected to. So that independently of the fracture of the bone, and of the possible disjunction of the articulations of the ulna, the success of the reparative process would be very problematical. In such cases the arm should be amputated. 4. The sooner amputation is performed, the greater is the propability of a favorable result. 5. The rapid and very extensive tumefaction of the limb constitutes a sufficiently certain criterion of the severity of the derangements which are propagated along its whole extent. When fractures are not detected in the diaphysis of the bone, some lesion of contiguity or continuity in the ulnar articulation must be suspected. 6. When the lesion does not seem severe enough to call at once for amputation, we must always be prepared for secondary occurrences which will unfit the limb for its functions. (In two of the author's cases, paralysis of the limb remained.) Still, conservative treatment in such cases should be attempted.—*Brit. & For. Med.-Chirurg. Rev.*, April, 1861, from *Omodei Annali Univ. di Med.*, vol. clxxiv.

